# Supplementary material for: The Deletion of LeuRS Revealed Its Important Roles in Osmotic Stress Tolerance, Amino Acid and Sugar Metabolism, and the Reproduction Process of Aspergillus montevidensis
Source: J Fungi (Basel). 2024 Jan 3;10(1):36. doi: 10.3390/jof10010036 (PMC10820851; doi:10.3390/jof10010036)
Supplement: Supplementary file 1 [file jof-10-00036-s001.zip › jof-2714259-supplementary.pdf]

Deletion of LeuRS revealed its important roles in osmotic stress tolerance, amino acid and sugar metabolism, and reproduction process of *Aspergillus montevidensis*

Xiaowei Ding, Wanting Liu, Kaihui Liu, Xiang Gao, Yue Liu

School of Food and Biological Engineering, Shaanxi University of Science and Technology, Xi'an 710021, China.

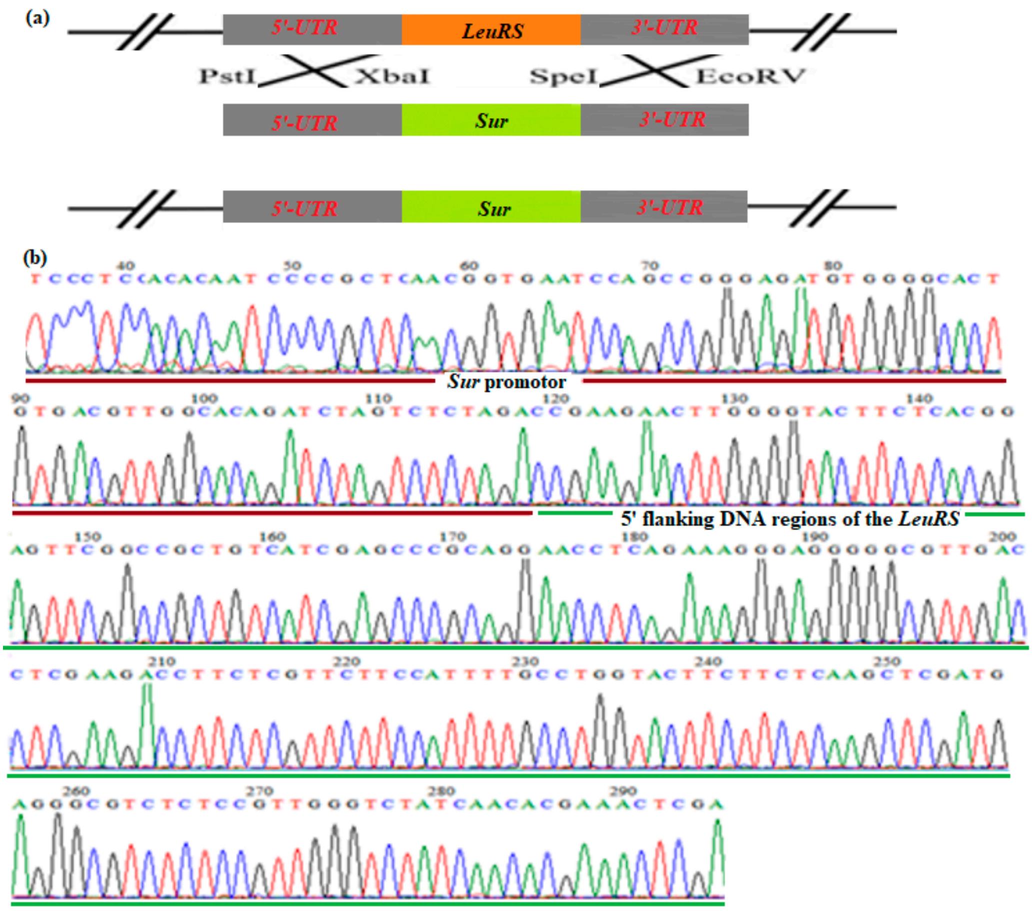

Figure S1. Knock-out of the gene *LeuRS* in *A*. *montevidensis* (WT). (a) The gene replacement strategy for the construction of the Δ*leuRS* strain. (b) Sequencing verification of Δ*leuRS* mutant of *A*. *montevidensis*.

Fig. S2 PCA of differential metabolites between Δ*LeuRS* mutants and WT strains treated with 1.5 M NaCl for 0h (C), 1h (SS), and 14 days (LS), respectively.

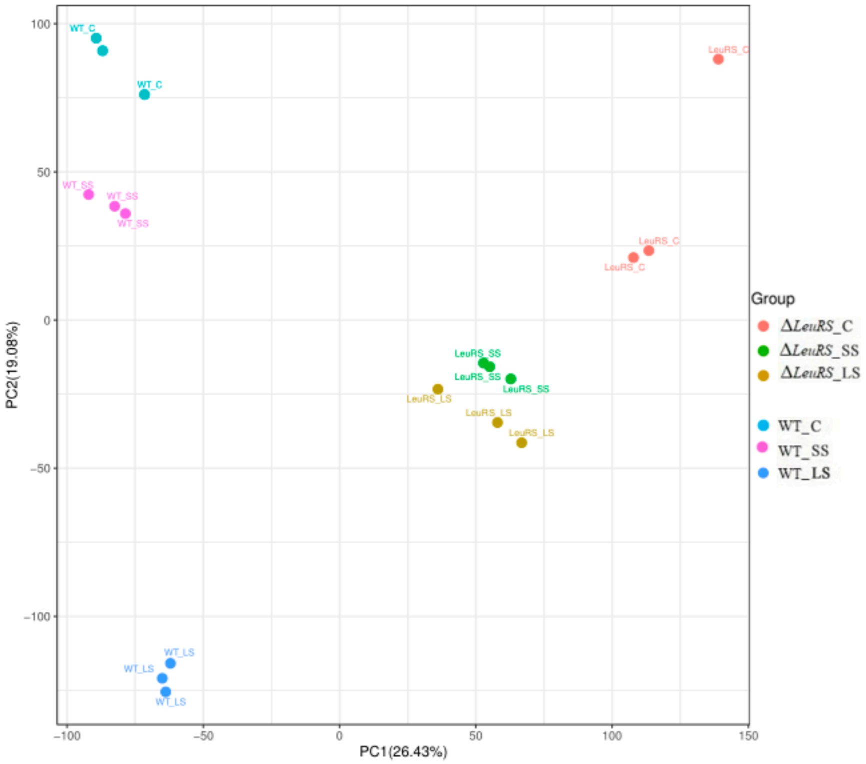

Table S1 The FPKM values of DEGs in *LeuRS* and WT strains treated with 1.5 M NaCl for 0h (C), 1h (SS), and 14 days (LS), respectively.

KEGG pathway	Gene ID	ΔLeuRS_C1	ΔLeuRS_C2	ΔLeuRS_C3	ΔLeuRS_SS1	ΔLeuRS_SS2	ΔLeuRS_SS3	ΔLeuRS_LS1	ΔLeuRS_LS2	ΔLeuRS_LS3	WT_C1	WT_C2	WT_C3	WT_SS1	WT_SS2	WT_SS3	WT_LS1	WT_LS2	WT_LS3	Putative functions
ABC transporters	Unig_1685	6.21	5.83	5.78	7.4	7.23	6.69	1.8	1.15	1.57	0.18	0.16	0.13	0.17	0.38	0	0.36	0.37	0.42	ABC transporter G family member 21
	Unig_4891	2.14	1.47	1.83	2.92	2.86	2.99	2.93	2.81	3.49	6.51	6.4	6.76	9.04	9.73	9.37	7.13	7.6	7.72	ABC a-pheromone efflux pump AtrD
	Unig_2816	16.12	16.48	16.2	18.15	18.58	18.4	30.92	30.58	30.81	64.98	67.39	66.55	60.37	59.93	60.14	49.4	47.49	51.26	Long-chain fatty acid import protein
	Unig_3061	0	0	0.32	0	0.19	0.19	0.57	0.15	0.33	1.21	1.45	1.27	0.42	0.32	0.18	2.14	2.11	2.23	ABC multidrug transporter Mdr1
	Unig_4393	2.95	3.39	3.3	2.1	2.04	2.61	80.62	79.55	83.43	62.73	59.17	60.77	97.21	99.03	96.17	102.33	104.38	104.29	ATP-binding cassette, subfamily G
	Unig_1016	0.3	0.35	0.24	0.58	0.73	0.62	4.5	4.93	4.44	7.86	7.29	7.43	12.28	11.83	11.76	19.25	18.4	19.23	ATP-binding cassette, subfamily G
	Unig_1056	2.42	2.23	1.91	3.09	2.75	2.54	25.97	24.88	24.74	94.19	97.26	95.33	54.38	55.14	55.49	11.18	10.24	10.87	ATP-binding cassette, subfamily G
	Unig_1160	2.03	2.17	1.83	2.26	2.91	2.03	41.7	38.61	39.98	127.9	127.48	131.72	64.49	69.5	68.53	9.22	9.16	9.16	ATP-binding cassette, subfamily G
	Unig_4149	0	0	0	0	0	0	0	0.42	0	0.21	0	0.61	0.47	0.26	0.2	0	0	0	ABC-2 type transporter
	Unig_3060	0	0	0	0	0	0	0	0	0	0.73	1.59	0	0	0	0	1.42	1.54	0	ATP-binding cassette, subfamily B
Nitrogen metabolism	Unig_3950	1.27	1.78	2.33	1.01	1.15	1.33	0.39	0.3	0.19	0.17	0.2	0.16	0	0	0.2	0.29	0.22	0	Carbonic anhydrase
	Unig_0422	29.39	26.56	28.3	30.08	27.21	27.63	42.25	42.13	42.61	78.83	80.15	80.05	97.91	97.22	98.81	92.48	96.48	91.62	Nitronate monooxygenase
	Unig_3764	0.81	0.92	1	1.07	1.04	0.98	14.35	12.62	13.58	19.39	18.47	18.49	13.64	13.22	12.8	3.29	3.03	3.38	Nitrate reductase
	Unig_2426	0.45	0.29	0.33	0.21	0.44	0.33	1.86	3.17	2.45	18.18	16.58	14.52	6.37	8.04	6.73	0.41	0.37	0.21	Nitrite reductase
Alanine, aspartate and glutamate metabolism	Unig_6877	85.01	86.99	89.35	103.37	107.99	102.55	208.34	201.72	204.03	44.5	40.33	44.87	21.38	22.3	24.69	97.46	103.08	99.36	Aspartate carbamoyltransferase
	Unig_3008	0.19	0	0	0.6	0.19	0.31	0.52	0.52	0.64	0.51	1.02	0.87	1.55	1.15	2.07	0.82	0.95	0.82	Adenylosuccinate synthase
	Unig_1305	9.1	9.38	8.31	10.61	11.36	11.11	10.96	10.9	10.54	64.18	61.64	63.71	58.85	60.25	60.7	56.17	57.56	55.18	Adenylosuccinate synthase
	Unig_1465	3.46	4.05	4.32	4.64	4.86	4.4	10.14	8.7	8.73	11.2	9.84	10.23	26.74	26.09	27.23	22.26	24.26	26.08	Aspartate aminotransferase
	Unig_1335	2.29	2.41	1.97	2.07	1.4	1.52	0.72	1.02	1.17	9.5	8.78	9.18	13.69	13.65	14.28	17.23	17.48	20.41	Succinate semialdehyde dehydrogenase
	Unig_1320	6.41	7.37	5.57	10.59	9.98	10.32	33.81	36.4	34.67	102.28	102.66	99.2	287.7	283.5	281.24	312.2	310.69	317.21	1-Pyrroline-5-carboxylate dehydrogenase
	Unig_6491	0	0	0.18	0.41	0	0.16	0	0	0.19	0.78	0.73	0.65	0.59	0.79	0.69	0.39	0.18	0.95	L-Amino-acid oxidase
Arginine and proline metabolism	Unig_5572	0.31	0	0	0.49	0.3	0.22	2.9	2.81	3.55	0.23	0.36	0	0	0.29	0	0.45	0.33	0.33	D-Amino-acid oxidase
	Unig_1647	109.43	104.77	108.53	103.44	104.39	102.5	36.82	37.85	37.12	1.06	1.02	1.24	0.67	0.86	0.68	5.34	4.7	5.35	Agmatine deiminase
	Unig_0832	0.46	0.38	0.2	0.34	0.31	0.48	5.7	4.31	5.35	3.85	4.61	4.38	1.02	1.44	1.31	0.59	0.49	0.74	Agmatinase
	Unig_0297	22.27	30.11	28.27	22.58	22.59	24.81	7.82	7.02	5.86	8.58	8.5	9.1	13.63	14.14	14.48	24.74	25.95	22.79	Amidase
	Unig_7057	69.12	69.64	67.64	71.04	69.03	68.62	306.87	315.04	308.56	63.24	61.13	61.35	53.86	55.44	56.99	109.38	103.88	102.11	Ornithine decarboxylase
	Unig_2559	69.09	69.44	66.38	65.09	67.52	65.56	150.56	147.95	150.16	406.46	416.13	409.88	440.34	453.18	439.18	1087.19	1080.53	1102.26	S-Adenosylmethionine decarboxylase proenzyme
Arginine biosynthesis	Unig_5890	0.31	0	0	0	0.46	0	3.58	5.85	4.38	0	0	0	0	0	0	0	0	0	Glutamine synthetase
	Unig_0121	37.01	35.53	35.47	34.37	33.58	32.48	22.74	22.09	20.45	39.92	41.41	39.89	63.67	62.14	66.44	183.49	176.43	175.17	Ornithine carbamoyltransferase
	Unig_4583	129.94	131.8	129.96	138.46	134.68	136.64	62.68	64.5	63.42	217.65	226.46	220.75	278.98	275.62	272.93	188.68	190.94	190.68	Glutamate dehydrogenase
	Unig_0069	51.55	51.08	51.26	57.58	57.47	56.57	73.05	72.24	72.13	230.79	235.68	235.51	284.77	283.92	283.1	226.86	225.77	223.7	Carbamoyl-phosphate synthase
	Unig_4934	26.44	26.8	26.22	27.35	26.23	25.53	66.42	65.54	66.1	263.19	269.84	269.39	352.14	348.92	353.47	306.1	308.74	310.49	Arginase
	Unig_4935	13.23	0	15.85	0	0	4.32	0	0	0	84.38	67.57	95.14	12.2	9.86	15.02	21.5	26.99	19.06	Arginase
	Unig_6346	0	0	0	0	0	0	0	0	0	68.29	77.76	58.56	32.87	36.47	52.26	51.31	57.42	27.23	Arginase
	Unig_0425	84.93	85.24	83.11	75.46	72.51	80.16	26.92	26.79	24.07	114.31	114.27	112.03	186.49	183.59	187.02	224.21	227.7	223.25	Argininosuccinate lyase
	Unig_1073	177.85	170.82	173.75	152.18	154.46	153.09	66.25	67.52	66.6	744.34	744.81	743.97	1244.15	1226.84	1255.54	270.83	269.43	267.98	Argininosuccinate synthase
	Unig_5058	0	0	15.5	0	0	0	0	0	0	49.36	51.5	57.53	0	8.99	9.84	0	0	0	Argininosuccinate synthase
	Unig_2828	0	0	0	0	0	0	0	0	0	12.72	32.21	12.36	0	11.59	15.84	0	0	0	Argininosuccinate synthase
Tryptophan metabolism	Unig_0813	33.98	32.92	33.87	39.37	38.72	39.16	60.21	61.11	58.68	37.25	37.77	36.04	42.24	42.31	41.78	57.31	57.99	58.64	Kynurenine aminotransferase
	Unig_0864	47.55	48.47	47.71	50.7	50.13	50.21	60.8	59.32	58.66	196.9	194.66	192.71	334.6	328.76	330.14	227.44	226.43	227.07	Kynureninase
	Unig_3544	0.39	0.23	0.41	0.08	0.45	0.33	0.31	0.62	0.41	1.34	1.22	1.24	0.52	0.6	0.79	1.88	1.4	2.06	Amidase
	Unig_3677	0	0	0.19	0	0	0	0.41	0	0.26	0.94	1.37	0.91	1.08	1.67	1.79	1.37	1.31	1.01	NADPH-cytochrome P450 reductase
	Unig_5590	0	0	0	0	0	0	0	0	0	0	0	0	0	0	0	1.45	1.56	1.71	Aminocarboxymuconate-semialdehyde decarboxylase
	Unig_0117	1.08	0.54	1.14	1.02	0.75	0.44	3.61	3.53	3.51	5.44	3.62	3.45	11.42	12.02	13.51	8.28	7.98	9.14	3-(3-Hydroxy-phenyl)propionate hydroxylase
Cysteine and methionine metabolism	Unig_0789	619.42	610.92	620.93	746.04	741.85	751.88	705.25	702.1	699.19	177.41	180.05	181.69	190.38	190.42	193.51	226.87	226.49	230.27	Cysteine synthase
	Unig_2764	0.22	0.12	0.11	0.08	0.12	0.12	0.34	0.6	0.44	0.91	0.64	0.74	0.59	0.79	0.54	1.97	1.96	2.1	Phosphoserine aminotransferase
	Unig_2160	6.28	5.12	6.6	3.44	3.36	4.4	2.5	2.77	3.67	6.94	5.51	5.87	7.63	7.91	7.06	14.62	15.25	14.92	3-Phosphoglycerate dehydrogenase
	Unig_2871	0.61	1.75	0.97	0.92	1.19	1.62	1.82	0.95	1.59	0.54	0.57	0.72	0.39	1.16	0.9	25.61	22.74	22.39	Threonine dehydratase

Sugar metabolism	Unig_3538	3.17	2.63	3.93	2.43	2.62	2.72	0.89	0.89	0.83	0.78	0.96	0.54	0.31	0.42	0.34	1.26	1.87	1.51	Sugar transporter STL1
	Unig_1097	7.33	6.9	7.58	6.86	7.32	6.8	12.79	12.12	12.07	18.11	18.22	18.93	14.13	14.26	14.2	15.69	15.27	14.44	Sugar transporter STL1
	Unig_0336	1.01	0.54	0.51	2.49	2.14	2.31	11.44	10.6	10.57	7.91	8.33	8.36	5.97	5.51	5.16	5.9	5.38	6.33	Sugar transporter STL1
	Unig_5073	1.3	1.4	1.34	1.48	1.58	1.56	24.68	24.03	23.45	131.07	131.94	132.13	72.53	75.8	72.07	4.29	4.12	4.48	Sugar transporter STL1
	Unig_4621	0	0	0	0	0	0	0	0	0	0.81	0.53	0.63	0.23	0	0.38	0	0	0	Sugar transporter STL1
	Unig_3134	0	0	0	0	0	0	0	0.19	0	0.57	0.59	0.49	0.37	0.54	0.3	1.4	2.15	1.42	Sugar transporter STL1
	Unig_5671	73.67	52.24	72.31	31.38	22.31	40.2	24.61	17.31	0	39.99	39.65	62.75	9.37	11.22	24.5	0	0	10.46	Enolase
	Unig_5333	0.56	0.86	0.7	1.54	1.23	1.51	129.06	131.83	130.87	10.44	10.29	10.49	13.8	11.44	13.63	6.17	6.35	6.58	Pyruvate decarboxylase
	Unig_1538	7.7	6.59	7.24	7.41	5.51	6.86	11.94	12.58	11.95	21.54	22.05	21.69	26.08	23.51	24.2	17.8	18.45	18.26	Aldehyde dehydrogenase
	Unig_3085	0.49	0.43	0.67	0.38	0.18	0.41	1.21	0.8	0.61	2.19	1.26	1.18	0.53	0.81	0.87	0	0	0	Hexokinase
	Unig_4585	1936.53	1917.73	1926.38	1753.92	1835.33	1823.24	2651.95	2665.76	2623.65	994.84	1010.66	1031.63	1131.71	1120.65	1148.46	775.09	788.79	784.67	Glyceraldehyde 3-phosphate dehydrogenase
	Unig_7022	199.79	200.24	200.09	220.78	226.01	228.23	318.53	317.48	320.36	307.8	305.17	304.68	208.14	203.68	208.96	78.23	80.57	79.99	Phosphoenolpyruvate carboxykinase
	Unig_4713	6.36	5.57	5.51	7.75	7.34	7.61	120.08	120.01	126	134.25	139.38	128.23	101.41	99.22	95.65	15.54	17.81	18.79	Alcohol dehydrogenase
	Unig_1841	1.35	1.1	1.15	1.02	1.21	1.38	0.39	0.71	0.41	17.11	16.34	16.12	22.33	22.67	23.34	87.94	89.65	87.06	Aconitate hydratase
	Unig_1392	13.21	12.84	12.8	12.34	13.1	12.53	0.56	0.22	0.3	0	0.4	0.31	0	0	0	0	0	0	Citrate synthase
	Unig_4759	1.19	1.21	1.06	0.83	0.78	1.07	0.28	0	0.27	0.32	0.45	0.23	0	0	0	0.56	0	0	Citrate synthase
	Unig_0380	2.13	2.02	1.94	1.34	1.64	1.94	27.39	27.98	28.33	67.09	69.13	68.23	38.14	40.45	36.2	8.94	7.69	8.53	Succinyl-CoA synthetase
	Unig_0160	282.81	275.64	284.5	279.65	271.52	271.76	151.03	150.66	149.64	48.02	46.64	47.39	42.07	42.35	43.67	28.13	28.35	28.49	Malate dehydrogenase
	Unig_3272	66.22	64.61	68.33	59.5	59.99	59.7	148.11	143.7	146.84	69.46	65.93	68.81	50.86	51.38	50.31	33.35	34.3	34.56	Ribulose-phosphate 3-epimerase
	Unig_1107	69.45	62.59	62.73	58.07	46.92	53.21	32.56	31.74	32.16	8.95	10.57	7.97	17.59	16.73	17.28	10.01	10.54	13.57	6-Phosphogluconate dehydrogenase
Glyoxylate and dicarboxylate metabolism	Unig_5896	0.45	0.35	0	0.24	0.22	0.22	0.62	0.3	0.5	0	0	0	0	0	0	0	0	0.24	Oxalate decarboxylase
	Unig_3313	5.5	5.29	5.77	6.23	7.23	6.01	83.92	78.34	79.5	34.63	34.97	35.34	35.38	36.98	35.7	15.52	15.75	15.09	Catalase
	Unig_0920	0.12	0.73	0.41	0.95	0.4	0.63	106.6	110.4	106.31	17.3	16.92	16.09	7.96	7.42	6.48	31.42	29.39	30.83	Catalase
	Unig_1315	0.67	1.32	0.84	0.7	0.71	1.22	35.03	32.56	36.14	8.95	8.75	9.75	14.45	12.96	13.86	15.15	13.3	14.12	Catalase
	Unig_2534	946.31	933	988.56	778.9	782.27	793.65	374.75	380.85	375.68	56.85	59.35	60.92	36.63	37.11	40.99	10.07	10.5	12.65	Glycine hydroxymethyltransferase
	Unig_1732	105.66	102.08	103.8	99.77	100.44	94.82	122.22	121.01	125.95	592.39	586.91	586.26	458.62	447.84	454.5	145.56	144.99	141.37	Malate synthase
	Unig_6438	16.95	17.71	16.65	16.2	16.03	15.88	31.27	30.26	30.01	96.48	98.2	98.31	92.47	89.02	92.58	46.89	47.27	45.34	Aminomethyltransferase
	Unig_3026	0	0	0	0	0	0	0	0	0.27	2.1	3.27	2.13	1.42	1.54	1.85	3.69	4.82	4.35	(S)-2-Hydroxy-acid oxidase
Linoleic acid metabolism	Unig_1220	16.85	14.31	14.63	13.43	14.8	13.84	4.59	5.74	5.66	3.04	3.49	3.54	3.84	3.73	3.28	7.98	9.41	10.46	Cytosolic phospholipase
	Unig_1243	12.81	12.69	12.59	12.95	13.29	13.24	40.21	40.92	39.47	31.51	32.93	31.67	46.31	46.25	45.68	25.55	24.78	24.06	Linoleate 10R-lipoxygenase
Propanoate metabolism	Unig_5640	7.55	7.05	7.84	7.35	7.8	7.57	10.53	10.98	10.76	29.71	29.79	29.97	29.45	28.29	27.18	86.33	84.37	85.52	2-Methylcitrate dehydratase
	Unig_3886	6	7.37	3.09	3.53	2.05	3.89	42.18	39.68	50.09	820.93	865.2	840.84	1417.66	1381.4	1361.28	15051.6	15348.7	15149.3	Methylisocitrate lyase
	Unig_1311	1.11	1.5	2.17	1.25	1.5	1.55	0	0	0	0	0.24	0	0	0	0.24	0	0.27	0.91	Methyl acetate hydrolase
Butanoate metabolism	Unig_2461	15.94	16.18	15.39	16.06	13	14.55	1.62	1.4	1.44	3.07	3.45	3.48	2.23	2.66	2.67	1.65	1.83	1.55	Acetolactate synthase
	Unig_0345	11.27	11.01	9.53	9.62	10.52	10.34	4.64	5.05	4.01	2.77	3.07	3	2.04	2.49	2.62	1.47	1.52	1.5	Tartrate dehydrogenase
	Unig_1954	0	0.63	0.3	1.41	0.26	0.26	0.63	0.27	0.4	3.4	4.12	3.4	8.88	9.36	7.86	2.09	2	2.6	Acetoacetyl-CoA synthetase

Transcription factors	Unig_0390	7.67	8.55	7.5	5.85	7.1	7.33	0.96	0.74	0.67	3.75	2.79	3.12	2.13	1.57	1.66	0.8	0.68	2.01	Zn(II)2Cys6(C6) transcription factor (nscR)
	Unig_3200	0.94	1.91	1.73	1.9	3.35	2.75	1.02	1.83	2.44	0.81	0.76	1.15	1.45	1.19	1.13	1.38	1.79	1.94	Zn(II)2Cys6(C6) transcription factor (Acr-2)
	Unig_4786	19.77	19.77	20.57	21.11	20.94	22.47	16.8	16.42	17.76	37.06	36.54	34.12	45.27	45.86	47.22	42.43	42.7	44.91	Zn(II)2Cys6(C6) transcription factor (AmdR)
	Unig_1580	10.06	9.69	10.6	15.51	14.78	14.21	19.67	21.24	18.17	41.11	39.94	40.28	44.06	45.41	49.06	30.94	32.96	33.87	Zn(II)2Cys6(C6) transcription factor (nosA)
	Unig_2785	1.34	1.45	1.65	1.69	2.22	1.55	2.93	3.33	2.73	15.36	17.98	16.29	13.84	14.88	14.61	18.83	18.18	18.9	C6-like factor-arginine metabolism regulation protein II (A)
	Unig_2463	0	0	0	0	0.44	0	3.58	5.05	4.34	4.03	3.99	4.94	3.12	3.76	3.56	1.13	0.87	0.96	Zn(II)2Cys6(C6) transcription factor
	Unig_4075	0	0	0	0	0	0	0	0.36	0	0.62	0.75	0	0.43	0.25	0.34	0.52	0.85	0.48	Zn(II)2Cys6(C6) transcription factor
	Unig_6788	179.08	180.41	170.5	144.97	142.08	145.72	117.79	116.03	113.26	434.41	438.51	430.45	305.67	312.17	310.98	118.04	120.11	121.91	Zinc knuckle transcription factor
	Unig_4524	20.4	19.53	19.26	22.92	22.94	23.14	16.79	16.23	15.65	56.58	57.54	58.5	82.72	85.86	84.35	58.65	58.95	59.27	C2H2 transcription factor (Swi5)
	Unig_2970	7.02	6.92	7.52	7.38	8.27	8.32	18.53	17.29	17.33	34.59	34.04	33.69	36.21	34.78	32.37	18.42	19.04	17.39	Zn-finger transcription factor
	Unig_7108	29.55	26.89	31.58	27.84	29.36	30.05	65.24	64.41	67.07	5.09	5.73	5.5	7.47	8.01	8.02	16.19	14.46	16.5	Conidiophore development (brlA)
	Unig_0484	27.94	25.41	26.34	29.36	29.65	28.4	55.65	55.01	56.1	56.73	55.73	56.12	61.01	64.78	63.65	71.26	67.99	70.77	Ste20
	Unig_2872	21.62	20.85	20.53	18.27	20.49	20.69	65.53	65.51	68.37	110.11	106.76	108.72	159.45	153.52	160.7	163.31	161.57	159.02	HOG1(SakA)
	Unig_5354	0.73	1.49	0.9	1.52	1.19	2.17	1.64	1.92	1.67	0.27	0.34	0.22	0.48	0.34	0.13	1.69	0.77	1.26	Nitrogen assimilation transcription factor (nirA)
Reproduction processes	Unig_6752	0.4	0.55	0.7	0.74	0.84	0.57	0.78	0.99	0.44	2.43	1.7	2.64	1.03	1.76	1.61	0.92	0.75	1.01	MAT1
	Unig_6340	33.51	33.04	35.47	31.42	31.1	29.85	17.93	18.28	16.09	44.03	40.61	42.68	44.09	46.71	45.56	50.09	49	48.88	VelB
	Unig_1820	16.49	17.85	17.3	25.81	23.28	24.36	51.9	53.78	53.08	49.22	50.95	49.13	63.6	62.36	65.16	120.68	120.43	124.47	VeA
	Unig_1807	5.69	5.23	4.51	7.5	7.26	7.93	6.36	6.56	5.53	14.31	14.69	15.2	33.55	33.51	33.3	38.06	41.93	40.04	VeA
	Unig_6796	12.24	11.76	11.68	9.24	10.12	8.52	9.92	10.47	10.33	41.61	41.53	40.86	14.24	13.98	13.51	5.48	4.9	5.33	wetA
	Unig_2325	7.31	7.53	7.36	7.26	8.09	8.44	30.59	30.79	29.17	29.03	29.51	30.14	16.4	16.17	15.98	32.22	31.97	33.73	VosA
	Unig_2929	341.52	325.76	319.96	253.43	256.84	245.74	335.29	344.39	338.16	36.84	34.19	36.37	54.42	43.01	42.23	5.33	5.78	6.56	Arp1
	Unig_3245	8.14	9.62	6.68	13.07	11.04	13.76	29.68	28	28.62	0.79	0.45	0.46	0.35	0.11	0.26	0.19	0.21	0	Arp1
	Unig_0880	3.12	2.45	3.48	3.7	2.79	2.3	1.01	1.07	0.97	3.57	3.32	3.4	3.27	3.77	4	0.55	0.29	0.29	nosA
	Unig_1761	12.77	12.44	12.75	13.69	13.25	13.27	11.38	11.56	11.02	14.42	14.78	14.3	24.87	25.94	25.5	39.49	38.47	38.58	sidC
	Unig_2490	1.37	1.83	1.36	1.77	1.45	1.49	3.05	2.1	2.8	9.67	10.04	9.31	7.9	8.26	7.71	3.01	3.24	3.23	ricA
	Unig_6886	1.77	1.66	1.61	2.02	2.64	2.14	17.31	17.07	17.64	4.03	2.82	4.13	2.79	3.35	3.43	24.42	24.26	25.54	LaeA

Table S2 Differentially metabolites of in compared groups of Δ*LeuRS* mutants and WT strains treated with 1.5 M NaCl for 0h (C), 1h (SS), and 14 days (LS), respectively.

Name	M1.VIP[2]	△LeuRS_C1	△LeuRS_C2	△LeuRS_C3	△LeuRS_SS1	△LeuRS_SS2	△LeuRS_SS3
L-Serine	1.9	12896160.78	14995819.52	18927249.51	38187560.49	32082912.52	37324957.09
Suberic acid	1.9	565032.457	667859.8646	688390.3987	4127207.427	5700078.492	4027447.175
4-Nitrophenol	1.9	330069.4621	292220.7773	280431.6499	157163.2777	169674.4011	123909.4296
Succinic acid	1.8	6216810.141	37382881.13	34453299.43	153653020.3	92287775.91	160694034.7
Uric acid	1.7	4317614.944	3834763.032	3768941.5	7647876.839	5554589.573	7689412.314
L-Histidine	1.7	1326255.324	3948027.152	5809552.795	10550023.84	8106397.215	10393183.64
Scopolin	1.7	657930.0307	893052.914	798669.6698	96786.35258	478268.7788	181620.4741
Gluconolactone	1.7	35448590.08	81789416.66	83028222.12	9140589.615	10690202.69	6428352.954
Uridine	1.6	1587757660	3463593724	3495775428	973546085.6	790414298.8	862602814.2
Carnosine	1.6	1811268.896	1867671.848	2002723.883	8904157.487	15999870.38	6576744.099
D-Sorbitol	1.6	2027259.95	5994074.483	4155150.103	7835358.395	7045867.453	7548843.999
Gentisaldehyde	1.5	5293570.75	32775429.39	28074789.52	456434.0044	349389.5677	736378.8
Azelaic acid	1.5	517566.6532	2378607.102	1557791.393	9237096.312	5028780.482	3635504.659
3-Methoxybenzenepropanoic acid	1.5	1585099.471	1167527.151	2613880.165	344625.9911	872561.396	703261.7887
Indoleacetic acid	1.5	390625.0467	1869188.804	1726180.826	3355345.224	2115214.119	2683276.087
2-Hydroxystearic acid	1.5	46769562.95	154640625.1	150516606.5	210314610.2	194527344.9	187879793.1
Mesylate	1.4	20638676.61	68661203.49	75126405.14	17614879.39	17306337.87	15931905.82
Deoxyuridine	1.4	47180518.54	81955773.25	87999260.78	123338063.3	84966617.41	141074564.6
3-Methylxanthine	1.4	9443645.629	13764802.36	7944586.117	12816994.48	14913316.82	15562111.38
4-Dodecylbenzenesulfonic Acid	1.4	77403127.45	285677194.4	247074327.7	273046670.2	413705157.9	386429838.4
Pyroglutamic acid	1.4	2060697.656	1735309.669	2234109.625	8959320.451	2096281.247	8835506.588
Methylsuccinic acid	1.4	2103069.011	3556520.067	2984324.047	8405423.268	3842646.39	5022306.65
L-Asparagine	1.4	3029148.501	6384595.459	6334419.379	8465745.022	6680256.311	7751373.427
PE(16:0/18:2(9Z,12Z))	1.4	273246348.3	1698675876	1342688115	270311876.6	310001156.7	231148702.3
Glycyl-glycine	1.3	1505890.144	4863180.163	4966566.684	7642475.837	4630274.066	8449401.148
Quinolinic acid	1.3	1135043.152	1672347.457	1054651.159	1941540.727	1736699.486	1512646.641

D-Mannose 1-phosphate	1.3	282915.2787	7777146.91	6991952.566	551724.5017	616218.9047	240743.0564
Adenosine monophosphate	1.3	529819.4124	13279064.29	11011816.86	1000917.763	1153613.199	420254.9967
Indole-3-carboxylic acid	1.3	1194208.282	5083568.367	4501750.481	6010085.05	6198404.232	5381146.838
Uridine 5'-monophosphate	1.3	218712.7044	10253250.23	9066237.216	924344.1078	575904.4927	247268.4771
4-Pyridoxic acid	1.3	431937.4106	648434.3123	669631.656	879066.4234	632653.0181	1071243.597
Methylmalonic acid	1.3	16964892.22	11183155.27	10010766.18	14845076.83	18522884.13	24106154.16
Isonicotinic acid	1.3	553530.84	415966.0883	433376.7469	370046.4783	423184.0824	318871.5152
2-Hydroxybutyric acid	1.3	4980961.097	32774513.86	31486803.72	6390106.287	7486421.078	6260973.715
Isocitric acid	1.3	70690.29517	3048956.871	1796171.652	164012.1088	22217.48234	60747.85706
Cosmosiin	1.3	16223.99111	1511946.947	912724.6656	10422.86989	130118.612	0
Oxoadipic acid	1.3	432896.1653	17501301.05	18240667.1	2249238.248	1787977.699	1694830.34
UDP-D-galactose(2-)	1.3	287015.2661	13494146.45	11580648.97	1647310.361	1389870.167	1049297.941
Gentisic acid	1.3	31831.75151	535320.1254	604568.5105	172671.3037	0	30294.06999
DL-Mandelic acid	1.3	305238.0603	2227050.956	1930054.788	494277.0442	456718.8845	487701.564
3-Hydroxyphenylacetic acid	1.2	5562892.53	12841981.95	13608522.89	19714538.26	11589619.05	18922803
Terephthalic acid	1.2	1042639.776	9600546.683	9532939.186	2245492.206	2126503.137	1751066.297
LysoPA(18:1(9Z)/0:0)	1.2	497836.0648	4413684.614	3847327.26	4663259.309	4606890.198	5687280.08
Prostaglandin D2	1.2	40693887.16	37283100.31	35523044.59	33287806.87	34636528.62	36868368.62
H-THR-PHE-OH	1.2	1050998.743	6733409.063	6336658.644	8886718.305	6596493.526	7562513.049
LysoPE(18:1(9Z)/0:0)	1.2	578337.7485	1987379.181	2775031.662	589834.3981	955806.0594	815095.5694
Gluconic acid	1.2	4812753.39	473515275.4	747450197.1	81954276.14	79046894.76	89366999.51
4-Hydroxyphenylpyruvate	1.2	29624634.58	254770312	252322434.4	75542213.47	56929827.58	73265047.16
Maleamic acid	1.2	1558367.361	212116.0393	497688.6875	1519135.94	1668631.555	1066568.223
o-Cresol	1.1	1533600.176	4385409.324	3922039.979	5839770.081	3235101.902	6260046.18
2-Hydroxycinnamic acid	1.1	3447533.9	24328036.43	28873719.79	28155551.54	26618041.2	36882043.83
Threonic acid	1.1	448220.8518	92778926.53	92925805.82	23981233.14	15270454.42	18223917.26
Riboflavin	1.1	2745476.061	1703297.842	1716570.368	3565457.118	2143013.81	2673347.655
Caffeic acid	1.1	1848373.316	4316747.327	4933931.917	2461689.966	1538529.443	2894841.336
Pyruvic acid	1.1	454464764.2	2371492272	2165562172	2702899659	2091682792	2749786204
Phenylpyruvic acid	1.1	17429735.41	16646160.23	20569898.34	16199650.42	30358879.59	58255693.82
16-Hydroxy hexadecanoic acid	1.1	29827401.31	108251214	111222516.4	149242030.9	101274247	117117510.8
Sweroside	1.1	128246.4575	861311.9193	776147.8401	139387.9039	262455.7439	405752.5795

trans-Aconitic acid	1.1	14616782.69	161466587	155945969.3	53970785.33	43614273.48	46123546.81
3-Furoic acid	1.1	1256839.078	16806328.94	16698447.03	5557206.268	4515485.282	4641079.506
Citraconic acid	1.1	7120615.549	69866175.23	67981255.57	24417199.18	20068653.45	20885769.7
Gentiopicroside	1.0	1381845.206	740934.6564	595298.9629	572685.4398	803226.3463	216563.4028
10E,12Z-Octadecadienoic acid	1.0	22091197162	38560225007	35615075069	28003722749	21119925239	26266020075
1H-Indole-2,3-dione	1.0	1122058.357	2536970.125	2839001.471	3315393.287	2174377.997	3522796.316
N-Acetyl-L-phenylalanine	1.0	0	5805569.027	5822325.552	1471332.428	1590517.399	1519751.751
L-Glutamic acid	1.0	15748482.64	176816039.1	166363544.9	66025169.6	45423573.83	57259602.52
D-Galactose	1.0	3486956.797	371617108.4	386050451.7	131748170.2	70465667.31	100742293.9
N,N-Dimethylguanosine	1.0	409711.9876	0	0	334809.426	840192.8922	144271.0101
Uracil	1.0	2566686.57	2597007.966	2802428.722	2486372.097	2515735.473	2642373.806
Saccharopine	1.0	2461218.762	46445651.67	44721150.52	18083360.45	13548840.15	12082222.65
D-Gluconic acid	1.0	2706822.904	26951629.81	29612987.03	11005905.85	11897012.98	6757238.061
N-Acetyl-glucosamine 1-phosphate	1.0	770664.6398	8726964.433	9233471.75	3697784.944	3122159.78	2466194.293

Name	M1.VIP[2]	WT_C1	WT_C2	WT_C3	WT_SS1	WT_SS2	WT_SS3
D-Galactose	1.2	106934699.6	114850795.1	119128125.7	9284319.433	8037669.519	10655948.58
Pseudouridine	1.2	12834982.54	12966686.22	14021591.74	4406988.09	4178358.973	4188981.985
Glutaric acid	1.2	31078876.31	31410476.59	34004629.97	9121959.059	10473239.57	10374495.76
L-Lactic acid	1.2	298012683.3	311370696.5	278415003	99242800.71	107406321.1	116332541.1
D-Ribose	1.2	146599128.2	140476403	154251450.4	51084757.53	58344512.95	55961950.94
Gentisaldehyde	1.2	94909559.68	97403012.38	87002883.84	15429238.18	14134329.54	156775.7799
p-Hydroxyphenylacetic acid	1.2	5688026.961	6116993.15	5054691.728	1314786.001	1094984.795	1251588.751
Hydroxypropionic acid	1.2	20260039.05	21299740.44	20910280.81	15840047.14	15167856.35	15457186.41
3-Methyladipic acid	1.2	378779.1495	425815.5715	123752.2393	7412918.717	5999185.511	6982838.093
Kynurenic acid	1.2	0	0	0	2709385.977	2344869.446	2588096.717
Succinic acid	1.2	5527222.261	6166969.382	7466564.344	211337505.8	165708494.9	200415240.7
Oxypurinol	1.2	165939761	173457337.1	169963011.1	116479874.8	99568528.1	108729884.2
Mesylate	1.2	55465856.34	53062541.31	54849988.45	24839370.76	14280230.57	15352910.6
Indoleacetaldehyde	1.2	8051908.878	9712474.023	8465774.733	3693755.861	3637506.353	3962597.247
Suberic acid	1.2	526510.7095	500355.1347	605333.2555	3731384.835	3619828.008	4822269.471
o-Cresol	1.2	8535686.275	7718726.945	8426993.443	18576470.36	15512823.41	17306729.18
Terephthalic acid	1.2	2687102.718	2666509.164	3290034.769	991239.6784	936270.3114	920893.1957
Deoxyuridine	1.2	19660768.19	18915100.64	24024779.62	5155412.775	6309942.878	6300019.202
4-Dodecylbenzenesulfonic Acid	1.2	322571980.5	316843803.7	366876592.2	191016243.7	186957871.9	173094898.1
Gluconolactone	1.2	36213445.86	43856493.51	44654528.06	16115080.46	15723140.44	11785858.9
Threonic acid	1.2	51859791.41	54766433.08	70843989.18	6329035.504	5217036.029	9412696.283
Fumaric acid	1.2	5733805.346	5837163.851	5448357.076	6909139.227	6709804.395	6745989.097
Hydrogen phosphate	1.2	52618583.53	57124422.08	58556016.17	39886302.78	37719936.24	35251446.29
Erythrono-1,4-lactone	1.2	7038770.501	5978879.737	7381735.868	2998647.517	3006003.663	3432115.419
L-Tryptophan	1.2	9047728.324	10858405.69	12097435.82	3355838.77	3120152.803	3715435.986
H-THR-PHE-OH	1.2	101776.6271	145024.272	107852.8211	700597.9451	540316.2945	774143.4765
Acetylleucine	1.2	6675387.767	6986542.18	5690864.096	3372311.959	3570513.414	3867601.071
L-Threonine	1.2	19948068.06	21897209.21	27998554.11	5227662.086	5631045.745	5436080.079
2-Hydroxystearic acid	1.2	17210519.25	15114443.8	20992144	3631026.369	0	3776869.585
Astilbin	1.2	0	0	54862.33736	205550.957	151413.0124	188763.339

α-Ketoisovaleric acid	1.2	160499192.6	153031819.8	204388524.1	65620313.09	64288667.92	64218421.57
N-Acetyl-L-alanine	1.2	3733694.91	3841629.46	3217088.856	2387834.739	2481252.799	2379640.458
Methylsuccinic acid	1.2	2365959.031	2636414.991	2586681.617	6815814.835	5145582.913	5687898.19
Scopolin	1.2	88967.58821	85450.90179	291567.5445	905715.4998	753597.8195	672094.1503
Sucrose	1.2	53737033.82	62256958.85	107483146.4	392324107.5	295433219.8	284702966.2
DL-Phenylalanine	1.2	2225587.076	2128955.714	2117267.437	3002017.338	2687107.483	2760267.381
Saccharopine	1.2	10078932.42	9985335.061	13300963.51	4277513.775	3656272.32	3875584.915
Phenylpyruvic acid	1.2	8121541.006	8163916.484	9781380.452	5185008.741	5015023.128	5357893.827
Prostaglandin D2	1.2	26247985.05	24061106.38	25307572.79	15973198.91	19624805.51	17024395.07
4-Hydroxyphenylpyruvate	1.2	11182658.94	12420991.2	18711627.45	0	0	0
Ribitol	1.2	7692631.066	6003965.13	7790281.603	3234704.676	3572413.518	3810085.852
L-Histidine	1.2	2708265.327	3513625.277	3659321.077	1462177.906	1613249.132	1480035.022
Xanthohumol	1.2	1743980.172	862779.7623	721695.3775	4245018.177	4082624.22	3167722.531
L-Serine	1.2	10025927.91	9666033.295	10507156.04	7362493.518	6610537.526	5365692.707
Isonicotinic acid	1.2	2833380.847	2505508.911	1994579.413	5660314.07	5055118.827	4375560.676
Caffeic acid	1.2	2473988.102	2515021.086	2597885.916	3808308.229	3464170.855	3249888.783
Uric acid	1.2	5060090.043	6328363.361	4481036.578	2396630.061	2341735.414	2216899.435
Isocitric acid	1.2	4117880.152	2668695.175	2284836.928	0	0	160900.602
N-Acetyl-glucosamine 1-phosphat	1.2	1035924.589	1084643.637	706468.5837	360916.5961	338116.2358	302668.662
L-Phenylalanine	1.2	19756241.85	24347959.78	30057548.76	8101473.165	9477218.904	7674405.955
Uridine	1.2	388969243.4	371080560.8	617460679.8	37322183.35	31248483.72	39023273.37
4-Hydroxycinnamic acid	1.2	0	172577.1562	614929.4573	3176759.603	2115358.748	2040382.014
Indoleacetic acid	1.1	13227703.29	14177333.47	12609810.56	23439400.06	18409441.85	22147701.32
Indole-3-carboxylic acid	1.1	1564515.141	1917172.982	1813860.686	2915699.44	2499219.918	2495009.786
L-Methionine	1.1	3988914.699	4237730.936	6116017.269	1271403.737	1335404.317	1697727.386
2-(Methylamino)benzoic acid	1.1	26636040.37	26575756.96	26101000.48	39403966.46	33099753.87	34396669.38
2-Hydroxycinnamic acid	1.1	9581205.78	10744332.5	12971659.49	6027686.516	6573304.356	4505570.455
16-Hydroxy hexadecanoic acid	1.1	15068725.1	25080317.64	18161703.64	5059351.471	5842003.031	6331305.987
3-Hydroxyphenylacetic acid	1.1	23517169.64	21057216.85	31581302.42	59044796.67	44112794.03	47268424.93
Quinolinic acid	1.1	39803782.26	29901099.92	34073270.26	68681470.36	66279725.52	51219382.96
Cholesterol sulfate	1.1	11880139.26	7465978.967	12156702.91	3120435.097	4691744.388	3678683.666

Dopamine	1.1	2981531.847	3028853.477	2823895.816	2446847.978	2443083.713	2686841.539
Oxoadipic acid	1.1	737715.6396	764473.1986	1355893.779	4462899.629	2987590.987	2710261.287
DL-Mandelic acid	1.1	705344.1155	829393.8586	711375.7785	198261.6978	472091.932	423088.3034
D-Mannose 1-phosphate	1.1	661048.527	726110.4933	1127873.216	237990.5302	204569.1818	305180.8962
Riboflavin	1.1	891193.203	828754.3556	1120609.984	2830986.581	2185477.853	1758628.66
2-Hydroxy-3-(4-hydroxyphenyl)pr	1.1	5081611.761	7252613.156	5989048.047	2595764.525	2430811.943	3885825.104
UDP-D-galactose(2-)	1.1	527364.022	506957.8333	655653.1203	1639445.535	1243446.59	1027888.447
Carnosine	1.1	3586532.358	4562086.007	10864530.11	14925238.81	14395858.91	14212352.9
Gentiopicroside	1.1	256836.2665	145927.4233	252362.7066	1217092.394	606374.6956	941076.5046
3-Methyluric acid	1.1	736629.091	858928.4181	663639.1882	2872319.725	1607509.553	2985872.948
Citramalic acid	1.1	1705282.598	1989594.373	1406190.626	990837.5099	1120085.984	1080282.023
(1R,4S,7S,9S,11R)-9-Tert-butyl-7	1.1	528979.7331	560146.3071	759423.2887	1089235.446	1062925.822	855366.7536
3-Hydroxymethylglutaric acid	1.1	53673678.24	56006023.29	55521545.12	51988911.76	46913772.63	48656010.53
N-Acetylhistidine	1.1	1193545.609	1435968.993	1558374.152	996985.1619	1013694.456	1028491.253
p-Cresol glucuronide	1.1	28116219.35	24709438.46	36343891.03	54528892.65	40842987.79	50902451.31
N-(2-Furoyl)glycine	1.1	4093969.362	3733104.016	2859197.892	6588893.702	6973862.115	4905942.026
D-Glycero-D-galacto-heptitol	1.1	5377739.491	4418343.652	4790718.919	8899440.991	6525530.423	7085635.722
10E,12Z-Octadecadienoic acid	1.1	12359484030	9134763996	20541716699	2860904239	3238407817	2964942728
Uridine 5'-monophosphate	1.0	445128.0966	548580.0165	1041807.142	111766.6168	84266.84812	80622.44751
Orotic acid	1.0	7273410.315	7758620.131	8975066.661	11026660.81	9636197.867	9730762.231
Gluconic acid	1.0	158574567.2	220976880.2	349463347.9	53523042.09	77921180.45	71316036.96
5'-Methylthioadenosine	1.0	1154183.811	763323.0675	3223106.305	3485376.658	4066155.924	4423035.002
Cosmosiin	1.0	532493.0213	766020.8383	1383861.736	98373.8656	83654.46753	149452.0495
Cytidine monophosphate	1.0	45277.24045	65184.96923	100098.1701	12431.2769	8857.627141	30442.53962
L-Glutamic acid	1.0	39028781.55	43300825.4	61139319.82	31327413.44	20914129.2	20085507.3
D-Gluconic acid	1.0	13448743.31	16137381.97	14147864.83	23882135.81	24255599.79	17387365.19
Malonic acid	1.0	50522460.44	115555454.3	125377625.9	22870173.17	25564528.24	35314016.02
Maleamic acid	1.0	649075.709	381538.5866	747327.2663	8223192.866	3832016.698	3369636.734
4-Hydroxyproline	1.0	4702438.344	5753123.271	8740989.298	3353605.652	2547122.301	2820617.294
Citraconic acid	1.0	36338980.5	28347754.05	45202049.12	23509466.87	22381301.41	23941165.26
trans-Aconitic acid	1.0	83988298	63270501.44	104340605.5	52069360.98	49592208.96	53215891.93

Name	M1.VIP[1]	△LeuRS_C1	△LeuRS_C2	△LeuRS_C3	△LeuRS_LS1	△LeuRS_LS2	△LeuRS_LS3
Carnosine	1.7	1811268.9	1867671.85	2002723.88	5787927.19	5193575.55	5364115.84
Succinic acid	1.7	6216810.14	37382881.1	34453299.4	126070237	149896941	149022785
Quinolinic acid	1.7	1135043.15	1672347.46	1054651.16	13648112.9	21601359.8	20621905.2
Kynurenic acid	1.6	88523.1346	7886145.83	248980.128	1450757.7	2508710.17	2229058.54
Uric acid	1.6	4317614.94	3834763.03	3768941.5	2573665.26	2827143.96	2947784.37
Azelaic acid	1.6	517566.653	2378607.1	1557791.39	4508062.36	4312163.42	4279676.27
Sweroside	1.6	128246.457	861311.919	776147.84	8583561.51	16862143	16185330.3
2-Oxovaleric acid	1.6	607792.389	1510120.87	1675659.1	2809382.6	3379266.94	3086834.4
Cytidine monophosphate	1.5	99242.2081	548579.472	673618.182	1144224.11	1918122.31	1648606.84
Gluconolactone	1.5	35448590.1	81789416.7	83028222.1	6083143.53	5646730.25	7045404.98
Astilbin	1.5	391483.07	587190.728	467731.57	210735.113	233952.606	290175.431
o-Cresol	1.5	1533600.18	4385409.32	3922039.98	21287641.9	55312539.2	48252827.7
Uridine	1.5	1587757660	3463593724	3495775428	326540443	610667440	577790110
Pyruvic acid	1.5	454464764	2371492272	2165562172	3561996263	5504248267	5311915491
2,3-Dihydroxybenzoic acid	1.5	0	3486909	3716014.42	11625493.4	29017577.1	22308439.9
3-Hydroxyphenylacetic acid	1.5	5562892.53	12841982	13608522.9	65351353.2	177846061	155485225
Indoleacetic acid	1.5	390625.047	1869188.8	1726180.83	2683773.02	3605351	3815870.55
L-Arabitol	1.5	1076220.24	2313451.94	3254646.97	5531026.55	8795346.56	5127599.81
Phenyllactic acid	1.5	12223645	130715983	70180938.2	212616987	555017791	487075792
Phenylpyruvic acid	1.4	17429735.4	16646160.2	20569898.3	26987221.1	49853704.2	39752746.2
Suberic acid	1.4	565032.457	667859.865	688390.399	9889434.39	4754147.19	3937260.5
α-Ketoisovaleric acid	1.4	629077582	3295495511	3203325610	4289272906	7019630110	6733522614
Deoxyuridine	1.4	47180518.5	81955773.2	87999260.8	38865835.7	30668333.8	35471215
5'-Methylthioadenosine	1.4	1238935	4173928.43	4721592.93	257514.873	343295.513	273583.437
Isonicotinic acid	1.4	553530.84	415966.088	433376.747	605172.804	1193632.52	988688.756
4-Nitrophenol	1.4	330069.462	292220.777	280431.65	97305.5965	197131.804	247520.47
Methylsuccinic acid	1.3	2103069.01	3556520.07	2984324.05	3610915.49	5292851.35	4626725.62
Gentisaldehyde	1.3	5293570.75	32775429.4	28074789.5	596712.113	1010025.87	661760.036
L-Histidine	1.3	1326255.32	3948027.15	5809552.8	5654907.43	9998828.47	9107620.58
D-Glycero-D-galacto-heptito	1.3	631887.775	7858637.88	5470823.51	16866105.2	75676324.7	43137608.5
PE(16:0/18:2(9Z,12Z))	1.3	273246348	1698675876	1342688115	73256378.2	159698509	77987337.3
3-Methoxybenzenepropanoi	1.3	1585099.47	1167527.15	2613880.17	1074644.51	286020.283	599138.07
4-Hydroxycinnamic acid	1.3	0	181671.22	203544.838	347132.595	1106650.61	563906.482

Scopolin	1.3	657930.031	893052.914	798669.67	563821.361	646757.454	633650.023
1H-Indole-2,3-dione	1.3	1122058.36	2536970.12	2839001.47	469899.736	1172061.44	993716.824
4-Pyridoxic acid	1.3	431937.411	648434.312	669631.656	396287.697	415720.699	420586.073
(1R,4S,7S,9S,11R)-9-Tert-b	1.3	1181038.86	1126914.28	942994.125	693495.857	731527.045	1017166.39
Pyridoxine	1.3	87149569.3	908177024	321684947	733751459	1400041698	1203595347
Pyroglutamic acid	1.3	2060697.66	1735309.67	2234109.63	7183874.35	3633214.37	3240762.84
4-Hydroxyphenylpyruvate	1.2	29624634.6	254770312	252322434	255977015	489805957	448166659
Mesylate	1.2	20638676.6	68661203.5	75126405.1	20243900.3	22317159.7	19445249.2
Indole-3-carboxylic acid	1.2	1194208.28	5083568.37	4501750.48	675052.663	1539438.24	1214964.95
Taurine	1.2	16051543.2	151642715	169796198	12234892.4	22526069.3	18274865.9
Glycylproline	1.2	4829857.98	15993193.4	17657449.4	2156099.56	5897101.81	5868548.65
Adenosine monophosphate	1.2	529819.412	13279064.3	11011816.9	341714.452	1056297	671240.222
Terephthalic acid	1.2	1042639.78	9600546.68	9532939.19	615934.865	2093867.1	1405057.77
Cosmosiin	1.2	16223.9911	1511946.95	912724.666	3606.10499	16564.1897	0
16-Hydroxy hexadecanoic a	1.2	29827401.3	108251214	111222516	94427762.7	220953271	194645947
Gluconic acid	1.2	4812753.39	473515275	747450197	4671123.89	23057683.5	24467866.7
DL-Phenylalanine	1.1	709196.754	2495294.47	2350437.5	2342741.49	3429465.55	3400629.95
Ribitol	1.1	3331529.57	14910906.4	13678766.9	14946237.5	31349671.5	18635777.7
2-Hydroxybutyric acid	1.1	4980961.1	32774513.9	31486803.7	7592396.16	7161070.62	6834623.28
Oxoadipic acid	1.1	432896.165	17501301	18240667.1	897387.226	2353131.35	2257987.6
N-Acetyl-glucosamine 1-pho	1.1	770664.64	8726964.43	9233471.75	1123310.66	1872875.71	1808467.98
Deoxyinosine	1.1	97164.5867	238330.514	228327.666	141326.591	97422.0995	74202.0822
Uridine 5'-monophosphate	1.1	218712.704	10253250.2	9066237.22	463743.284	1732834.47	1234119.08
Succinic acid semialdehyde	1.1	18740974.6	66198621.2	62917292.4	59181652.9	86835721.5	97750496.4
Uracil	1.1	2566686.57	2597007.97	2802428.72	2542809.09	2533638.85	2516977.56
D-Mannose	1.1	48717266.7	286221600	277295464	55123852.8	89058691.6	78449298.4
2-(Methylamino)benzoic aci	1.1	14687282.9	55293566.2	58138831.2	49025932.8	93688131.4	80970067.9
Isocitric acid	1.1	70690.2952	3048956.87	1796171.65	16199.221	460386.871	184177.019
trans-Aconitic acid	1.1	14616782.7	161466587	155945969	25771121.6	38641126.1	33459201.7
L-Malic acid	1.1	6440014.2	8501182.76	8307370.98	13800414	23377757.1	7989979.82
Citraconic acid	1.1	7120615.55	69866175.2	67981255.6	12142250.6	17384275.9	15553679.3
3-Furoic acid	1.1	1256839.08	16806328.9	16698447	2564924.64	3990186.23	3275364.85
LysoPE(18:1(9Z)/0:0)	1.1	578337.749	1987379.18	2775031.66	753009.338	1012623.81	517831.465
L-Serine	1.1	12896160.8	14995819.5	18927249.5	13505898.5	30877700.5	29620020.7
2-Hydroxystearic acid	1.0	46769562.9	154640625	150516607	112604196	300820495	237856362
L-Asparagine	1.0	3029148.5	6384595.46	6334419.38	4735184.46	11670341.3	10193626.5

Hypoxanthine	1.0	8804717.77	8733086.68	29143831.1	20037272.8	31985898.3	29592664.4
Methylmalonic acid	1.0	16964892.2	11183155.3	10010766.2	7888735.5	6669165.74	11694254.1
D-Gluconic acid	1.0	2706822.9	26951629.8	29612987	6465911.05	7136890.83	8078249.71
Allopurinol-1-ribonucleoside	1.0	488604.157	273111.713	340185.388	143378.152	349445.273	226257.85
UDP-D-galactose(2-)	1.0	287015.266	13494146.5	11580649	772490.685	3753548.47	2600907.68
Riboflavin	1.0	2745476.06	1703297.84	1716570.37	1817297.95	1526966.96	1075314.93
Glutaric acid	1.0	23394587.9	44496829.4	47762673	21456672.9	29322984.7	30196544.6
Aminoadipic acid	1.0	463299.045	5858547.7	5349615.82	596604.681	1904761.2	1659397.23

Name	M1.VIP[1]	WT_C1	WT_C2	WT_C3	WT_LS1	WT_LS2	WT_LS3
H-THR-PHE-OH	1.2	101776.6271	145024.272	107852.8211	9021279.839	8987296.658	8761052.105
Oxypurinol	1.2	165939761	173457337.1	169963011.1	15366311.1	15222051.83	15260457.77
Mesylate	1.2	55465856.34	53062541.31	54849988.45	20709432.52	20238482.6	20390650.18
DL-Phenylalanine	1.2	2225587.076	2128955.714	2117267.437	8629094.588	8136595.186	8617995.145
Pyridoxine	1.2	242206841.1	219922318.8	229008736.6	1102626791	1152795466	1187821466
D-Ribose	1.2	146599128.2	140476403	154251450.4	20440989.74	17585450.15	15372962.44
Sweroside	1.2	195633.1696	110211.4684	160694.4081	2029411.484	1999836.191	1852024.449
Kynurenic acid	1.2	0	0	0	2044742.928	2206726.334	2293709.316
Gentisaldehyde	1.2	94909559.68	97403012.38	87002883.84	262758.6423	305300.3513	277242.9915
Glutaric acid	1.2	31078876.31	31410476.59	34004629.97	3958062.192	3351910.655	2659188.788
m-Coumaric acid	1.2	11067258.26	10304002.34	9799577.645	408369.192	107406.0412	427178.2776
N-Acetylhistidine	1.2	1193545.609	1435968.993	1558374.152	4091730.862	4416583.995	4316904.91
Pseudouridine	1.2	12834982.54	12966686.22	14021591.74	5460004.423	5520770.977	5804686.146
Glyceraldehyde	1.2	16867855.31	16162584.21	20455777.88	43773057.91	45170504.85	43920235.05
Methylsuccinic acid	1.2	2365959.031	2636414.991	2586681.617	5696334.264	5249831.279	5707437.897
Zearalenone	1.2	0	0	0	1601133.491	1873680.558	1940004.187
2,3-Dihydroxybenzoic acid	1.2	334489785.5	316766714	252470186	1087161487	1198434168	1250260096
Quinolinic acid	1.2	39803782.26	29901099.92	34073270.26	173845720.7	206666569.2	190653428.8
Indole-3-carboxylic acid	1.2	1564515.141	1917172.982	1813860.686	302522.2923	256814.3395	227972.1401
D-Mannose 1-phosphate	1.2	661048.527	726110.4933	1127873.216	2829658.778	2786720.171	2891457.001
Succinic acid	1.2	5527222.261	6166969.382	7466564.344	136587684.9	166997591.9	176040398.2
Gluconolactone	1.2	36213445.86	43856493.51	44654528.06	7035596.578	8619305.821	7648242.761
UDP-D-galactose(2-)	1.2	527364.022	506957.8333	655653.1203	5800142.422	7557049.275	6406411.48
Caffeic acid	1.2	2473988.102	2515021.086	2597885.916	3477945.757	3435599.444	3261958.678
Prostaglandin D2	1.2	26247985.05	24061106.38	25307572.79	12245062.82	9433057.66	7986254.913
4-Pyridoxic acid	1.2	740908.3622	545165.1154	504027.0865	1517672.296	1740632.426	1629758.632
2-Hydroxystearic acid	1.2	17210519.25	15114443.8	20992144	0	0	0
Isonicotinic acid	1.2	2833380.847	2505508.911	1994579.413	11004934.09	13800413.83	11139394.45
N-(2-Furoyl)glycine	1.2	4093969.362	3733104.016	2859197.892	22558298.61	19675017.25	17095934.89
Citraconic acid	1.2	36338980.5	28347754.05	45202049.12	89969237.42	100673277.7	93425638.74
Xanthohumol	1.2	1743980.172	862779.7623	721695.3775	5389341.074	5350741.584	4591208.607
Dopamine	1.2	2981531.847	3028853.477	2823895.816	2353457.511	2333667.007	2330746.276
3-Furoic acid	1.2	9011130.985	6761836.408	11288653.87	22355176.91	25081538.75	23491564.81

2-(Methylamino)benzoic acid	1.2	26636040.37	26575756.96	26101000.48	62426591.06	75244256.53	77911030.51
Cytidine monophosphate	1.2	45277.24045	65184.96923	100098.1701	805182.2312	583267.8421	697550.1093
Uric acid	1.2	5060090.043	6328363.361	4481036.578	327889.5146	191869.874	282862.2161
α-Ketoisovaleric acid	1.2	160499192.6	153031819.8	204388524.1	26252730.83	24564634.61	26516300.62
trans-Aconitic acid	1.2	83988298	63270501.44	104340605.5	210001087.2	242862821.9	219763047.9
2-Ketobutyric acid	1.2	22156454.77	24137313.27	26554627.55	43666993.5	45294375.59	52168745.57
L-Methionine	1.2	3988914.699	4237730.936	6116017.269	10752584.85	10017726.11	9797840.642
p-Hydroxyphenylacetic acid	1.2	5688026.961	6116993.15	5054691.728	3137360.005	3163680.347	3437704.836
Succinic acid semialdehyde	1.2	42142333.95	34585413.55	54777358.28	93490014.77	96326574.15	86441499.07
D-Mannose	1.2	10211896.06	10182247.29	7143916.955	17548159.72	16744420.19	18802030.55
N6-Methyladenosine	1.2	569795.7563	510157.9171	1598989.504	3622169.147	4214236.429	4650192.233
N,N-Dimethylguanosine	1.2	2101757.992	314751.1531	593173.7879	5208768.909	6576954.719	5725023.626
Taurine	1.2	5484320.869	4838376.575	11107628.89	19966992.83	21460144.99	21949671.87
Dulcitol	1.2	12137903.9	11333520.01	8749364.032	18827784.07	18758464.41	21418348.66
Succinic anhydride	1.2	2291583.84	2375933.325	4597190.191	7715925.323	8188862.716	8137545.286
Saccharopine	1.2	10078932.42	9985335.061	13300963.51	18010567.67	18356009.4	17991844.05
Fumaric acid	1.2	5733805.346	5837163.851	5448357.076	6845857.328	6729915.091	6477077.463
3-Methyladipic acid	1.2	378779.1495	425815.5715	123752.2393	3090178.942	4048473.223	5159703.713
Cholesterol sulfate	1.2	11880139.26	7465978.967	12156702.91	937832.8624	1137719.908	1352241.159
N-Acetyl-glucosamine 1-phosphate	1.2	1035924.589	1084643.637	706468.5837	1649297.57	1703997.129	1669108.07
Deoxyuridine	1.2	19660768.19	18915100.64	24024779.62	11467764.98	10309086.82	11109680.83
4-Hydroxyphenylpyruvate	1.2	11182658.94	12420991.2	18711627.45	0	0	0
L-Serine	1.2	10025927.91	9666033.295	10507156.04	13684416.26	12852587.85	14805429.5
Hydantoin-5-propionic acid	1.2	8657569.301	8503230.346	7161593.974	5048662.078	3464719.049	4001586.354
Terephthalic acid	1.2	2687102.718	2666509.164	3290034.769	1482335.555	1740419.069	1434302.942
Acetylleucine	1.1	6675387.767	6986542.18	5690864.096	4048591.3	4101529.488	4337776.443
16-Hydroxy hexadecanoic acid	1.1	15068725.1	25080317.64	18161703.64	2633827.031	2510358.218	2518412.279
Deoxyguanosine	1.1	243220.1305	257291.8198	230058.4958	167905.4298	147520.0886	185685.0682
4-Nitrophenol	1.1	196284.616	249407.6144	296923.1189	497116.1676	691554.0197	618806.6294
2-Oxovaleric acid	1.1	1894126.795	1156970.999	2227029.829	3851838.93	3923403.068	3378074.598
D-Pantothenic acid	1.1	81306845.65	82499739.96	78731070.28	96003048.04	110289573	106118476.6
Phenyllactic acid	1.1	14766612.47	16691858.82	16232935.42	22054484.62	20158386.3	24228136.23
o-Cresol	1.1	8535686.275	7718726.945	8426993.443	10785083	9753024.345	11322224.94
Indoleacetaldehyde	1.1	8051908.878	9712474.023	8465774.733	6253629.411	4182917.923	5379521.676
3-Methylxanthine	1.1	693836.322	188935.9903	849477.2023	2087659.412	1602915.432	2631649.146

Uridine	1.1	388969243.4	371080560.8	617460679.8	130314387	118791415	118597131.6
L-Lactic acid	1.1	298012683.3	311370696.5	278415003	246312646.6	259119305.1	253245897.6
Alantolactone	1.1	1136298.517	1207825.652	337090.1861	20368523.41	37867193.22	19142133.3
10E,12Z-Octadecadienoic acid	1.1	12359484030	9134763996	20541716699	1932440330	1455116579	1250822314
Riboflavin	1.1	891193.203	828754.3556	1120609.984	1274924.223	1451450.232	1276381.981
4-Dodecylbenzenesulfonic Acid	1.1	322571980.5	316843803.7	366876592.2	263511963.2	245038908.4	183214834
Citramalic acid	1.1	1705282.598	1989594.373	1406190.626	1104601.24	1026120.61	1122802.61
N-Acetyl-L-phenylalanine	1.1	1114698.402	1356973.119	1206121.446	1514178.787	1448574.496	1591325.635
L-Asparagine	1.1	2380840.248	2911934.285	2492246.927	3325823.088	3437195.887	3053437.13
Ribitol	1.0	7692631.066	6003965.13	7790281.603	5465936.412	4836588.916	5065536.765
Sucrose	1.0	53737033.82	62256958.85	107483146.4	119152795.4	164171918.4	150278084
3-Methoxybenzenepropanoic acid	1.0	729975.3485	1052558.218	843021.3988	1523882.208	3097967.781	3012464.615
L-Histidine	1.0	2708265.327	3513625.277	3659321.077	4880567.94	4300418.875	4172160.941
Hydroxyisocaproic acid	1.0	61119589.26	61906214.65	64196795.43	41057398.92	47513926.79	20019274.69
Lactitol	1.0	686505.8561	650474.5525	895808.6592	1264868.154	1927593.407	1252752.754
Maleamic acid	1.0	649075.709	381538.5866	747327.2663	9528002.372	3915814.014	4554094.298
2-Hydroxycinnamic acid	1.0	9581205.78	10744332.5	12971659.49	8687577.884	5710150.152	7014098.138
Mannitol	1.0	20081675.45	16923456.79	13428999.59	30184957.03	29118328.99	21383976.44
Adenine	1.0	2818497.442	3941205.757	3169761.82	1821053.74	2231163.64	2470567.133
D-Gluconic acid	1.0	13448743.31	16137381.97	14147864.83	9017612.608	12736968.75	9691892.889
L-Sorbose	1.0	11677049.11	12005290.04	6955931.023	13936918	15855155.72	16511891.08
N-Acetyl-L-alanine	1.0	3733694.91	3841629.46	3217088.856	2935065.411	3146330.259	2936889.712
Oxoadipic acid	1.0	737715.6396	764473.1986	1355893.779	1427611.444	1797592.509	1587025.677

Table S3 KEGG pathways of metabolites of Δ*LeuRS* mutants and WT strains.

Common metabolites in LeuRS_SS/LeuRS_C and WT_SS/WT_C	LeuRS_SS/LeuRS_C (log2 FC)	WT_SS/WT_C (log2 FC)	KEGG pathways	Common metabolites in LeuRS_LS/LeuRS_C and WT_LS/WT_C	LeuRS_LS/LeuRS_C (log2 FC)	WT_LS/WT_C (log2 FC)	KEGG pathways
D-Mannose 1-phosphate	-3.7	-1.7	Fructose and mannose metabolism	D-Mannose	-1.9	1.1	Fructose and mannose metabolism
D-Galactose	-1.6	-3.5	Galactose metabolism	Gluconic acid	-4.8	-1.1	Pentose phosphate pathway
Gluconic acid	-2.6	-1.7	Pentose phosphate pathway	Gluconolactone	-4.1	-2.3	Pentose phosphate pathway
Gluconolactone	-3.7	-1.4	Pentose phosphate pathway	L-Serine	-0.3	0.6	Glycine, serine and threonine metabolism
L-Glutamic acid	-1.5	-0.9	Alanine, aspartate and glutamate metabolism	Glutaric acid	-1.3	-3.1	Lysine degradation
Carnosine	1.4	1.3	Histidine metabolism	DL-Phenylalanine	0.1	2.1	Phenylalanine, tyrosine and tryptophan biosynthesis
L-Histidine	0.8	-1.0	Histidine metabolism	Quinolinic acid	2.9	2.6	Tryptophan metabolism
Saccharopine	-1.4	-1.4	Lysine degradation	Kynurenic acid	-0.6	11.8	Tryptophan metabolism
Indoleacetic acid	0.5	0.8	Tryptophan metabolism	4-Hydroxyphenylpyruvate	0.8	-14.2	Tyrosine metabolism
Isocitric acid	-4.6	-5.6	Tryptophan metabolism	Citraconic acid	-2.0	1.5	Valine, leucine and isoleucine biosynthesis
Oxoadipic acid	-2.9	1.9	Tryptophan metabolism				
4-Hydroxyphenylpyruvate	-1.8	-14.3	Tyrosine metabolism				
Citraconic acid	-1.6	-0.6	Valine, leucine and isoleucine biosynthesis				
Unique metabolites in LeuRS_SS/C	LeuRS_SS/C (log2 FC)		KEGG pathways	Unique metabolites in LeuRS_LS/LeuRS_C	LeuRS_LS/LeuRS_C (log2 FC)		KEGG pathways
N-Acetyl-L-phenylalanine	-1.6		Phenylalanine metabolism	Pyruvic acid	1.0		Alanine, aspartate and glutamate metabolism
2-Hydroxybutyric acid	-2.2		Propanoate metabolism	Glycylproline	-2.1		Glycine, serine and threonine metabolism
Gentisic acid	-2.9		Tyrosine metabolism	Aminoadipic acid	-1.8		Lysine degradation
				4-Hydroxycinnamic acid	2.2		Tyrosine metabolism
				3-Hydroxyphenylacetic acid	2.9		Tyrosine metabolism
Unique metabolites in WT_SS/WT_C	WT_SS/WT_C (log2 FC)		KEGG pathways	Unique metabolites in WT_LS/WT_C	WT_LS/WT_C (log2 FC)		KEGG pathways
Sucrose	2.2		Starch and sucrose metabolism	D-Mannose 1-phosphate	1.9		Fructose and mannose metabolism
L-Lactic acid	-1.4		Glycolysis / Gluconeogenesis	Glyceraldehyde	1.5		Pentose phosphate pathway
D-Ribose	-1.3		Pentose phosphate pathway	Sucrose	1.1		Starch and sucrose metabolism
4-Hydroxyproline	-1.1		Arginine and proline metabolism	Dulcitol	1.0		Galactose metabolism
L-Methionine	-1.6		Cysteine and methionine metabolism	D-Ribose	-2.9		Pentose phosphate pathway
L-Threonine	-2.0		Glycine, serine and threonine metabolism	3-Methyladipic acid	3.9		Alanine, aspartate and glutamate metabolism
Glutaric acid	-1.6		Lysine degradation	L-Methionine	1.3		Cysteine and methionine metabolism
L-Phenylalanine	-1.5		Phenylalanine, tyrosine and tryptophan biosynthesis	N-(2-Furoyl)glycine	2.6		Glycine, serine and threonine metabolism
Kynurenic acid	12.0		Tryptophan metabolism	2-Ketobutyric acid	1.1		Glycine, serine and threonine metabolism
Indoleacetaldehyde	-1.1		Tryptophan metabolism	N-Acetylhistidine	1.8		Histidine metabolism
L-Tryptophan	-1.6		Tryptophan metabolism	Citraconic acid	1.5		Valine, leucine and isoleucine biosynthesis
4-Hydroxycinnamic acid	3.3		Tyrosine metabolism	m-Coumaric acid	-4.9		
p-Hydroxyphenylacetic acid	-2.1		Tyrosine metabolism				
